# The Roles and Pharmacological Effects of FGF21 in Preventing Aging-Associated Metabolic Diseases

**DOI:** 10.3389/fcvm.2021.655575

**Published:** 2021-03-31

**Authors:** Junbin Yan, Yunmeng Nie, Jielu Cao, Minmin Luo, Maoxiang Yan, Zhiyun Chen, Beihui He

**Affiliations:** ^1^The First Affiliated Hospital of Zhejiang Chinese Medical University, Hangzhou, China; ^2^Key Laboratory of Integrative Chinese and Western Medicine for the Diagnosis and Treatment of Circulatory Diseases of Zhejiang Province, Hangzhou, China

**Keywords:** fibroblast growth factor 21, aging, metabolic disease, glycometabolism, pharmacology, lipometabolism

## Abstract

With the continuous improvement of living standards but the lack of exercise, aging-associated metabolic diseases such as obesity, type 2 diabetes mellitus (T2DM), and non-alcoholic fatty liver disease (NAFLD) are becoming a lingering dark cloud over society. Studies have found that metabolic disorders are near related to glucose, lipid metabolism, and cellular aging. Fibroblast growth factor 21 (FGF21), a member of the FGFs family, efficiently regulates the homeostasis of metabolism and cellular aging. By activating autophagy genes and improving inflammation, FGF21 indirectly delays cellular aging and directly exerts anti-aging effects by regulating aging genes. FGF21 can also regulate glucose and lipid metabolism by controlling metabolism-related genes, such as adipose triglyceride lipase (ATGL) and acetyl-CoA carboxylase (ACC1). Because FGF21 can regulate metabolism and cellular aging simultaneously, FGF21 analogs and FGF21 receptor agonists are gradually being valued and could become a treatment approach for aging-associated metabolic diseases. However, the mechanism by which FGF21 achieves curative effects is still not known. This review aims to interpret the interactive influence between FGF21, aging, and metabolic diseases and delineate the pharmacology of FGF21, providing theoretical support for further research on FGF21.

## Introduction

Energy metabolism generally refers to the release, transfer, and utilization of energy in the process of biomass metabolism. Energy metabolism is mainly related to glucose, lipids, and proteins, among which lipometabolism and glycometabolism are particularly important. Lipid metabolism refers to digestion, absorption, synthesis, and decomposition of lipids with various related enzymes. Glucose metabolism is responsible for the formation and storage of glucose with the help of insulin (1). There is an interactive influence between glucose and lipid metabolism (2). Insulin resistance causes hyperinsulinemia, which inhibits lipolysis, increases lipid synthesis, and causes excessive lipid accumulation. Abnormal lipid metabolism, especially the accumulation of heterotopic lipids, promotes decreased insulin sensitivity in adipose tissue. The balance of energy storage and release, also known as energy homeostasis, is crucial for overall health and even survival (3). A long-term metabolic imbalance will lead to excess lipid accumulation and further contribute to obesity, non-alcoholic fatty liver disease (NAFLD) and type 2 diabetes mellitus (T2DM), known as aging-related metabolic diseases (4, 5). Adipose tissue dysfunction and excessive lipid accumulation are the basis of the pathogenesis of metabolic disease (6).

Aging is characterized by a deterioration in homeostatic process maintenance over time, leading to functional decline and increased risk for diseases. Thus, energy homeostasis is gradually disrupted with aging, and the risk of energy metabolism-related diseases increases (7). Some studies have revealed compelling evidence that energy metabolism has a crucial interaction with anti-aging regulation (8, 9). Selective elimination of aging cells can ameliorate several aging-dependent energy metabolic diseases (10, 11).

Fibroblast growth factor 21 (FGF21) is a peptide hormone synthesized by multiple organs and regulates energy homeostasis (12, 13). As a FGFs family member, FGF21 is crucial because it can directly improve lipid and glucose metabolism in cells and delay cellular aging (14). Thus, the association between FGF21, energy metabolic diseases, and aging has recently attracted increasing attention. We illustrate this systematically in this review.

## Connection Between Aging and Metabolism

Cellular aging is an irreversible state of cell cycle arrest induced by various stressors, including telomere dysfunction, genotoxicity, and oxidative stress (15). Telomere shortening, which occurs after cell divisions, is a common cause of internal cellular aging. After several divisions, cells may activate p53, p21, and pRb pathways due to telomere shortening, promoting growth arrest and cell aging. Cellular aging is a complex process with dual functions, which are both beneficial and harmful to health. Aging helps clear damaged cells and is involved in tissue recovery during injury or acute stress. Senescence-associated secretory phenotype (SASP) secretes chemokines and cytokines (such as IL-1B and MCP-1) to attract immune cells and clear aging cells (16). However, consistently, excessive SASP induced by cellular aging will accumulate too many aging cells, causing insufficient tissue regeneration (17). Aging affects multiple organs, mainly those with high metabolic demands such as liver, heart, and brain (18). Therefore, aging may be a major risk factor for many metabolism-related diseases (19) and closely related to metabolism.

Metabolic dysregulation (including mitochondrial dysfunction) is one of the aging hallmarks (7, 20). Interestingly, aging-associated pathways (such as AMPK and mTOR), which are significant targets of anti-aging interventions, either directly regulate or intersect with metabolic pathways (21). With the development of technology, metabolomics has received increasing attention and quantitatively analyzes all metabolites in organisms (22, 23). Through metabolomic analysis, it was found that the existence of specific metabolic intermediates is an anti-aging intervention target, and even more directly a biomarker of aging (24). These hub metabolites represent nodes in the metabolism and aging network that play a crucial role in regulating information flow between metabolism and signaling pathways to control aging.

Nicotinamide adenine dinucleotide (NAD^+^), a common hub metabolite, is a crucial center connecting metabolism and aging. As an essential cofactor, NAD^+^ plays a central role in regulating energy metabolism, including glycolysis, fatty acid oxidation, and tricarboxylic acid (TCA) cycle, and it can also mediate DNA repair and gene expression (25). Recent studies have highlighted the various roles of NAD^+^ in aging. A metabolomics study quantified the plasma level of NAD^+^ in people aged 20 to 87, showing that the levels of NAD^+^ decreased significantly with age (26). Reduced levels of NAD^+^ are associated with several aging-related diseases, including metabolic diseases, cancer, and neurodegenerative diseases (27). Additionally, the dietary administration of NAD^+^ precursors has been shown to increase NAD^+^ levels in aging tissues, thereby improving aging and aging-related diseases (28, 29). Research has directly proven that NAD^+^ can directly control SASP and regulate cellular aging (30). The above studies have confirmed that the metabolite NAD^+^ can directly regulate cellular aging. Moreover, not only NAD^+^, nicotinamide adenine dinucleotide phosphate (NADP), and αKG can regulate cellular aging (31–33).

Scholars have also directly confirmed that aging is affected by metabolism through animal experiments. Mlekusch et al. found that controlling the exercise of mice, leading to a decrease in metabolic levels, will cause mice to age and shorten their life span (34). Even as early as over a century ago, it was discovered that the metabolic rate is related to aging: Rubner discovered that smaller animals have a higher resting metabolic rate. Based on this, a “rate of living hypothesis” was created, which believed that exhausting a limited number of metabolic events would lead to death (35, 36). This theory is not only applicable to mammals. The metabolic rate of birds is twice that of mammals of the same size, but their average life span is approximately three times that of mammals that match their body mass (37).

Therefore, there is no doubt that metabolism and aging are closely related, but whether FGF21 is related to it or whether it can be used as a connection point between metabolism and aging is still unknown. We will elaborate below.

## The Relationship Between FGF21, Metabolism, and Aging

### FGF21 and Its Family

**Figure 1:**
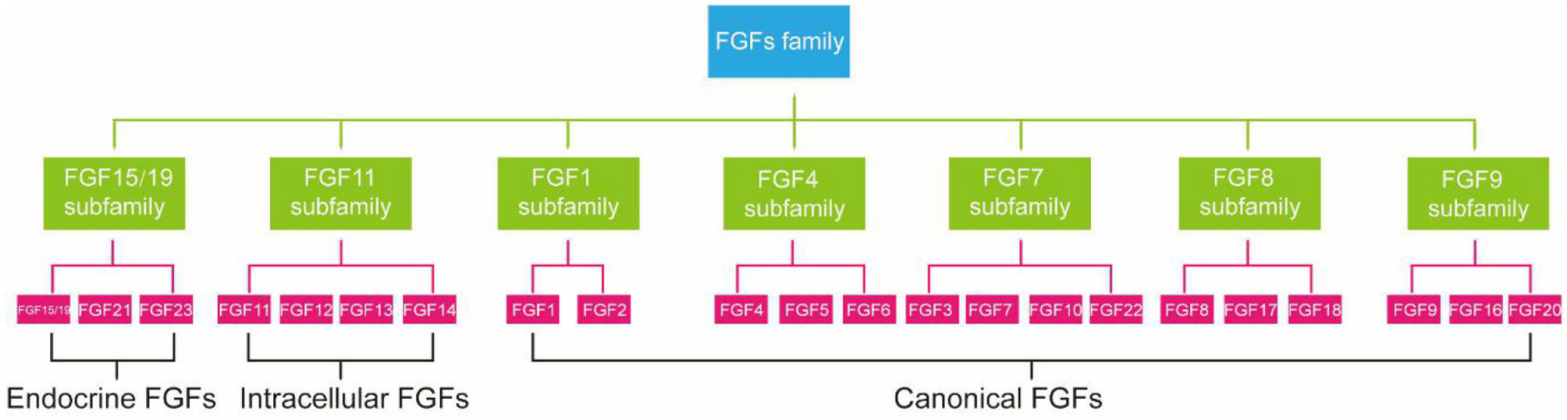
Subfamilies of FGFs confirmed by phylogenetic analysis.

FGFs family consists of 23 members but only 18 FGFR ligands. Four family members (FGF11, FGF12, FGF13, and FGF14) cannot bind to FGFR are more correctly referred to as FGF homologous factors (38). FGFs are effective regulators of cellular aging. Not only that, mutations in FGFs have been linked to many metabolic diseases, including atherosclerosis, NAFLD, and diabetes (39). According to phylogenetic analysis, FGFs family members can be further divided into seven subfamilies (Figure 1) (40). FGF21 is a member of an endocrine FGF subfamily, which includes FGF15/19 and FGF23. FGF21 can circulate and diffuse freely in tissues as an endocrine factor because of the lack of heparin-binding domain (41). FGF21 is expressed in many tissues, including liver (42), adipocytes (43), brown adipose tissue (BAT), pancreas (44), gastrointestinal tract, brain, skeletal muscle, and heart, and it directly regulates the metabolism and aging of peripheral tissues (12). Next, we will introduce the relationship between FGF21 metabolism and aging.

### FGF21, the Hub Linking Metabolism and Aging?

In 2005, FGF21 was first used as a novel metabolic regulator (45). As a coordinator of energy metabolism in multiple organs (especially liver and fat), FGF21 can regulate adipogenesis, glucose uptake, and cellular insulin sensitivity (12, 46–48). The level of FGF21 is elevated to promote the oxidation of free fatty acids (FFAs) and inhibit lipogenesis in the liver to supply energy when glucose levels are low or caloric restricted (49). In an animal experiment, Inagaki et al. found that FGF21 expression increased 28-fold in the livers of mice after 12 h of fasting. Increased FGF21 expression will stimulate ketogenesis in the liver and promote lipolysis in white adipose tissue to provide energy for activities (50). In addition to mice, in the fasting state, the level of FGF21 in human serum will also increase rapidly within a few hours to promote lipolysis (51). Proper administration of FGF21 also effectively improved insulin sensitivity and hepatic glucose uptake in obese mice (52). Studies have found that long-term administration of FGF21 to genetically obese mice will ameliorate fasting hyperglycemia via increased glucose uptake and improved hepatic insulin sensitivity (53). Lack of FGF21 in mice evokes insulin resistance and promotes gluconeogenesis and liver glucose production (54). In addition to liver and adipose tissue, the expression of FGF21 in skeletal muscle also has important metabolic functions. FGF21 could improve muscular dystrophy and atrophy through metabolic pathways (55, 56).

In addition to acting as a metabolic regulator, FGF21 can also improve aging. There are clear indications that the effect of FGF21 in preventing aging may be related to the thymus; overexpression of FGF21 can prevent aging-related changes, such as retarding thymus degeneration to prevent thymus weakness, improving immune system, and hopefully extending human life expectancy in the future (57). With aging, tissue autophagy is reduced, disrupting tissue ability to maintain protein homeostasis, thus accelerating the aging process (57). FGF21 can stimulate adiponectin secretion in fatty tissues, thus improving autophagy in target tissues to play an anti-aging role (58). Compared with normal mice, animal experiments show that fasting-induced FGF21 overexpression in mice and slowed aging (59). Transgenic overexpression of FGF21 significantly extended the life span of mice without reducing food intake or affecting NAD^+^ metabolism (60).

In summary, FGF21 mainly regulates aging by metabolism. It is crucial to clarify the mechanism or pathways of FGF21 in regulating metabolism and aging.

### The Mechanism by Which FGF21 Regulates Aging Through Metabolism

FGF21, a new type of endocrine hormone, is primarily produced by liver (61). Klotho proteins include α-klotho and β-klotho, of which β-klotho is an essential part of the FGF21 receptor complex and necessary for promoting high-affinity binding to its homologous FGF receptor (62). FGF21 signals through a receptor complex composed of fibroblast growth factor receptor 1 (FGFR1) and the coreceptor β-klotho, both required for FGF21 signaling and then activates downstream genes, to exerts its effect (63, 64). Studies have found that, as an endocrine messenger, FGF21 could induce hormonal responses in other tissues, such as the secretion of adiponectin from fat tissue and corticotropin-releasing hormone (CRH) from the hypothalamus, to maintain metabolic homeostasis (65). In addition, endocrine FGF21 can stimulate the secretion of digestive enzymes from pancreatic acinar cells, which require signaling through a tyrosine kinase receptor complex composed of an FGF receptor and β-Klotho to enhance the digestion of food in stomach (66). FGF21 can also indirectly maintain metabolic homeostasis by activating downstream pathways (67). In cultured adipocytes, FGF21 could regulate metabolism by activating MAPK and downstream ERK1/2, which triggers the activation of GLUT1 and glucose uptake (68). In liver, FGF21 positively controls the PI3K/AKT, insulin-like growth factor 1 (IGF-1), and mTOR pathways to maintain metabolic homeostasis (69).

AMPK, the downstream protein of FGF21, consists of three subunits (AMPKα, AMPKβ, AMPKγ), each with multiple phosphorylation sites, which can regulate lipid metabolism (70). AMPK phosphorylates sterol regulatory element binding protein-1c (SREBP1c) at Ser372, inhibiting the proteolytic cleavage of the precursor SREBP1c to mature SREBP1c, thereby inhibiting steatosis in diet-induced hepatic insulin-resistant mice (71). AMPK can also indirectly inhibit the expression of SREBP1c by reducing mTORC activity, thus decreasing liver lipid content (72) or phosphorylating adipose triglyceride lipase (ATGL), to stimulate TG lipase activity and activate lipolysis (73).

Amazingly, AMPK is highly conserved in eukaryotes, giving them the ability to expand their lifespan (74). By activating AMPK, FGF21 may delay aging and extend mammals' lifespan (75). Increased longevity has been observed in transgenic worms expressing the modified AMPK-γ subunit (76, 77). Overexpression of a single AMPK-α subunit in the fat body also extended the life span of fruit flies (78).

As a pro-longevity kinase, AMPK can also prevent cellular aging by activating downstream pathways (79–81). AMPK is associated with some downstream pathways involved in controlling aging, such as rapamycin complex 1 (mTORC1), nuclear factor kappa-B (NF-κB), and sirtuin-1(SIRT1). Low levels of inflammation promote aging (82). AMPK may extend longevity by inhibiting the NF-κB pathway and NF-κB-mediated inflammatory response (83). Activation of mTORC1 will inhibit autophagy and accelerate aging, while FGF21 exposure can inhibit the activation of mTORC1 in the liver resulting in anti-aging effects (84). AMPK can directly activate SIRT1 (75, 85, 86). After being triggered, SITR3 will mediate autophagy to anti-aging (87). FGF21 regulates mitochondrial biogenesis by activating PGC-1α through the FGF21-AMPK-SIRT1 pathway (88). FGF21 also stimulated the expression of PGC-1α in mouse liver (89) and human dopaminergic neurons (90, 91). Improved mitochondrial function and activation of PGC-1α play a crucial anti-aging role (92). AMPK-SIRT1 axis is also connected to several other aging-linked targets, such as p53 and HIF1α (93). Therefore, it is clear that anti-aging effects can be achieved by activating AMPK, but whether FGF21 can inhibit aging by upregulating the expression of AMPK requires further experimental proof.

**Figure 2:**
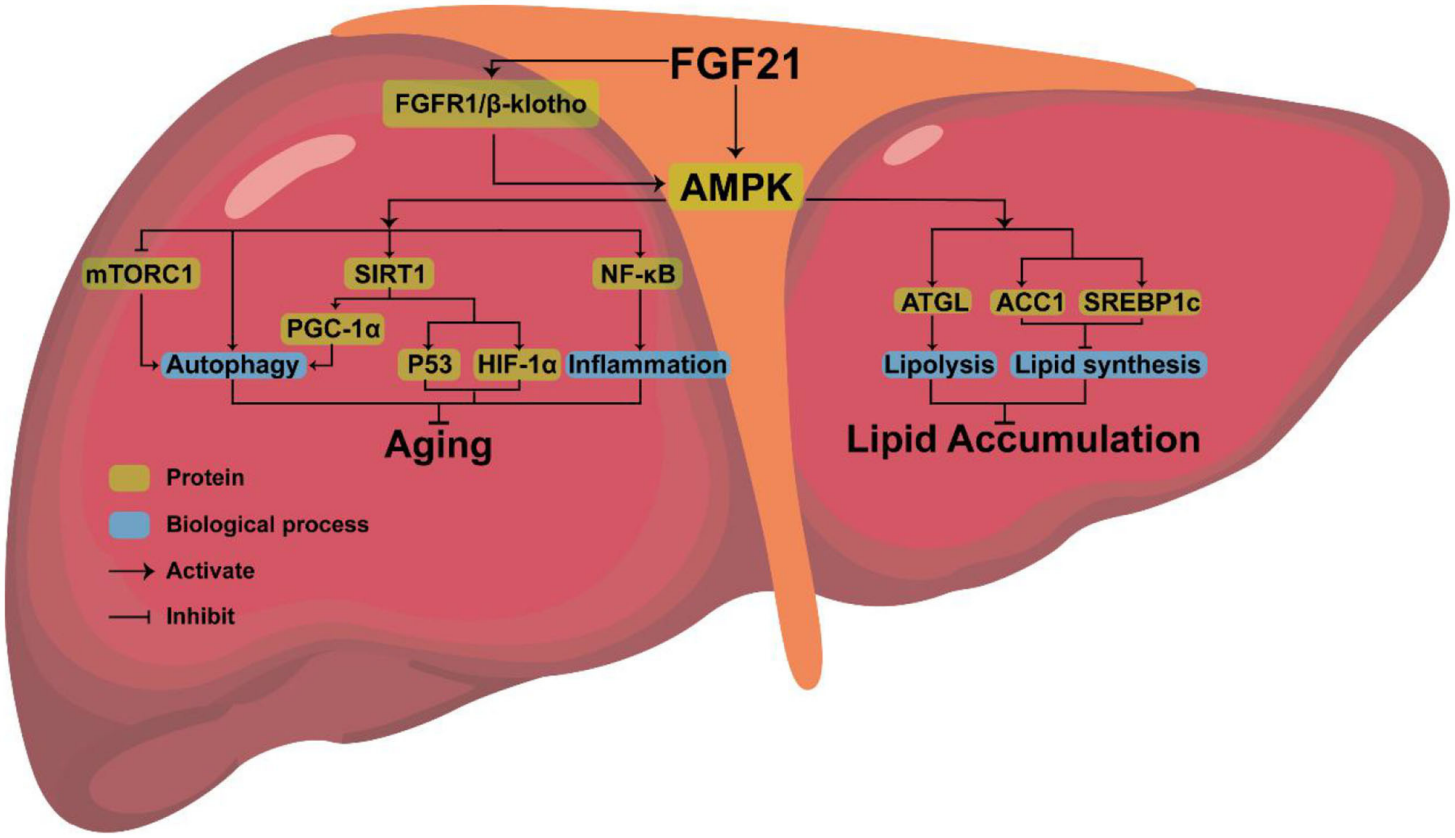
The possible mechanism of FGF21 simultaneous regulation of aging and metabolism.

Combined with the above analysis, we believe that AMPK, the downstream protein of FGF21, may be the key to FGF21 simultaneous aging and metabolism regulation (Figure 2).

## The Actions of FGF21 in Aging-Related Metabolic Diseases

The possible mechanism by which FGF21 simultaneously improves metabolism and cellular aging has been described above. However, the relationship between FGF21 and various aging-related metabolic diseases has not yet been clarified; thus, in the following, we will focus on the relationship between FGF21 and obesity, T2DM, and NAFLD.

### FGF21 With Obesity

Obesity is a global epidemic metabolic disease that affects infants, children, and adults (94). Over the past three decades, the global prevalence of obesity has nearly doubled; the number of obese and overweight people has exceeded that of underweight people globally (95). Obesity is associated with several complications, such as cardiovascular disease, hypertension, dyslipidemia, NAFLD, insulin resistance, hyperglycemia, T2DM, and is related to neurodegenerative diseases, and cancer (96, 97). Thus, it is urgent to pay more attention to obesity.

Studies have found that FGF21 is nearly negatively related to obesity. FGF21 transgenic mice fed high-fat, high-carbohydrate (HFHC) food are resistant to weight gain and obesity (45). Activation of FGF21 will cause weight loss in obese patients. For example, recombinant FGF21 treatment can reduce weight without changing the food intake of diet-induced obese (DIO) mice (98). After FGF21 intervention for 6 weeks, the weight and fat content of DIO mice were significantly lower than those of mice that did not receive FGF21 treatment. Lipid homeostasis and hepatic steatosis in DIO mice in the FGF21 treatment group were significantly improved. An animal experiment also found that chronic treatment with recombinant FGF21 reduced serum and liver TGs levels in diet-induced obese mice by inhibiting sterol regulatory element-binding protein-1 (SREBP-1), a transcription factor critical for fat formation, to achieve the goal of weight loss (98). Potential application of FGF21 as an anti-obesity molecule has even been approved in a study (99), in which exogenous FGF21 was administered.

Interestingly, FGF21 can promote weight loss, and FGF21 analogs also have the same effect. The FGF21 analog Fc-FGF21 protein (RG), results in weight gain inhibition in diabetic mice (100). FGF21, an FGF21 mutant used in combination with other weight-reducing drugs or exercise, could effectively cause weight loss in db/db mice (101). Recombinant murine FGF21 and leptin coadministration can also reduce the weight of mice (102). As a result, FGF21 is currently considered a potential target for the treatment of obesity.

### FGF21 With T2DM

Diabetes mellitus (DM) is defined as a metabolic disease characterized by hyperglycemia due to insulin secretion, insulin action, or a combination of both. T2DM, one subtype of DM, accounts for 90–95% of diabetic cases (103). World Health Organization (WHO) estimates that 347 million people worldwide have DM, of which 90% are T2DM (104). T2DM is characterized by insulin resistance, a relative lack of insulin, and hyperglycemia. Complications of T2DM include heart disease, stroke, diabetic retinopathy, and kidney failure (105). Because of its pervasiveness and dangerous effects, T2DM deserves more attention.

Some studies have found that FGF21 is an emerging T2DM treatment target. FGF21 can increase the therapeutic benefits of antidiabetic compounds such as metformin, glucagon/glucagon-like peptide 1 (GLP1) analogs, and thiazolidinedione (TZD) (106). The direct use of FGF21 can also reduce plasma glucose and TGs to near-normal levels in diabetic patients and animals. The experiment found that administering human recombinant FGF21 for 6 weeks can significantly improve fasting plasma glucose, TGs, insulin, and glucagon in diabetic rhesus monkeys (107). Interestingly, although human recombinant FGF21 has an excellent effect on lowering plasma glucose, it will not cause a hypoglycemic crisis. Human recombinant FGF21 can also improve the lipoprotein profile, including increasing high-density lipoprotein cholesterol, lowering low-density lipoprotein cholesterol, and significantly decreasing weight. Studies have also found that FGF21 can protect islets from glycolipid toxicity and cytokine-induced apoptosis and increase the insulin content of pancreatic β cells, which helps maintain glucose homeostasis in T2DM mice (108). Taken together, these results all support the idea that FGF21 is feasible for treating T2DM.

### FGF21 With NAFLD

NAFLD is one of the most common liver diseases globally, and it affects 25% of the population globally and 8% of children (109, 110). The disease is characterized by accumulating TGs in hepatocytes with little or no alcohol consumption (111). The NAFLD spectrum includes non-alcoholic fatty liver (NAFL) in the early stage, non-alcoholic steatohepatitis (NASH) in the middle stage, fibrosis in the late stage, and liver fibrosis associated with cirrhosis, liver failure, and even hepatocellular carcinoma (HCC) (112). Moreover, NAFLD is predicted to be the most frequent indication for liver transplantation in Western countries by 2030 (113).

There is growing evidence that aging is a vital risk factor for the occurrence of NAFLD (114, 115). Studies have found that cellular aging can induce mitochondrial dysfunction and reduce fat metabolism, resulting in excessive accumulation of hepatocytes and fatty degeneration (116). Mitochondrial dysfunction contributes to increased reactive oxygen species (ROS) (117, 118). Excessive ROS will lead to abnormal inflammation of the liver, leading to the deterioration of NASH. In aged mice fed a high-fat diet, age-related mitochondrial dysfunction further promotes oxidative stress, leading to worsening of NAFLD (119). In an animal experiment, rats were divided into obesity-prone and obesity-resistant groups. The former group showed more severe steatosis and significantly increased mRNA levels of p16 and p21, cellular aging-related genes, in the liver (120). *In vitro* studies found that the lack of cellular aging-related gene p53 in primary cultured hepatocytes could reduce the level of apoptosis and steatosis (121). Liver biopsies from patients with NAFLD also showed a significant increase in p53 expression in the liver compared to normal controls (122). These experimental results all confirmed that the overexpression of aging-related genes could promote hepatocyte steatosis, suggesting a crucial relationship between aging and NAFLD.

NAFLD can be improved by taking FGF21 as a therapeutic target, which is crucial for regulating cellular aging and energy metabolism. Some studies found that administration of FGF21, FGF21 analogs, or adenoviral delivery of FGF21 will reduce hepatic steatosis in diverse rodent models of NAFLD (123–125). In diet-induced obese mice, FGF21 or FGF21 analogs also decreased the expression of lipogenic genes, including stearoyl-CoA desaturase-1(SCD1), fatty acid synthase (FASN), and sterol regulatory element binding transcription factor 1 (SREBF1) (126). Inhibition of the expression of the lipogenic genes can effectively inhibit the synthesis of lipids and promote lipolysis. Acting as an autocrine agent, FGF21 can also activate genes that protect against oxidative stress to treat NAFLD. Many animal experiments support this conclusion. FGF21 also protects mice from acetaminophen-induced hepatic oxidative damage (127). In obese diabetic mice, FGF21 treatment reduces hepatic oxidative damage and lipid peroxidation (128). Moreover, in wild-type mice, FGF21 increases transcription of the oxidative stress response and antioxidant genes, including superoxide dismutase 2 (Sod2), glutathione peroxidase 1 (Gpx1), sirtuin (Sirt1), and forkhead box transcription factor 3 (Foxo3) (129).

## Potential Therapeutic Pharmacology of FGF21

FGF21 has beneficial pharmacological effects on T2DM, obesity, and NAFLD, and a large number of preclinical studies have been reported (99, 130). A single injection of FGF21 in obese (ob/ob) mice and DIO mice, can cause a rapid reduction in blood glucose and plasma insulin levels. At the same time, glucose tolerance and insulin sensitivity improve (131). Nevertheless, the application of natural FGF21 as a drug has encountered some obstacles. Because it requires intravenous administration and the circulation half-life (0.5–2 h) is too short, it may be due to rapid renal clearance and proteolysis. Thus, this has led to the development of FGF21 analogs and FGF21-receptor agonists.

LY2405319 (Eli Lilly and Co.), an FGF21 analog, was the first analog to be applied in obese patients with T2DM (132). It can improve dyslipidemia, reduce plasma insulin and body weight, and increase adiponectin levels. Nevertheless, only a tendency to decrease glucose was observed. This study shows that the treatment of LY2405319 is generally well-tolerated. PF-05231023 (Pfizer Co.) consists of two recombinant FGF21 molecules fused into the antibody fragment, which is a long-acting FGF21 analog that is can be administered once a week. For T2DM patients with hypertriglyceridemia who received a single dose of PF-05231023 (0.5–200 mg), dose-dependent decreases in triglycerides were observed. In addition, total cholesterol and low-density lipoprotein cholesterol decreased, while high-density lipoprotein cholesterol increased in the high-dose group (133). Another study with PF-05231023 reported a direct effect of FGF21 in the absence of weight loss (134). Recently, two clinical trials showned that pegbelfermin (BMS-986036), a PEGylated long-acting FGF21 analog (Bristol-Myers Squibb Co.), can be administered once a week. Subcutaneous injection of pegbelfermin for 12 weeks in obese and T2D patients can improve dyslipidemia, increase adiponectin and reduce the N-terminal level of fibrosis biomarker type III collagen propeptide (PRO-C3) without causing changes in HbA1c (135). A phase IIa clinical trial confirmed the efficacy of pegbelfermin in the treatment of NASH. Patients in this trial received subcutaneous pegbelfermin once a week for 16 weeks (136). The results showed that liver fat content in patients with NASH was significantly reduced and well-tolerated. Efruxifermin (AKR-001) is an FGF21-fc analog that has a sustained effect on insulin sensitivity and lipid metabolism in patients with T2DM. Short-term adverse reactions are limited, but more research is needed to study potential long-term safety issues (137).

In addition to FGF21 analogs, the FGFR1/β-klotho complex (FGF21 receptor agonists, FGF21RAs) opened a new door for aging-associated metabolic diseases, which have been tested in non-human primates (NHPs) and humans (138, 139). Thus far, it is unclear whether FGF21 receptor agonists have therapeutic advantages over FGF21 analogs. The first FGF21RA is C3201, an 18 kDa bispecific avimer peptide with high affinity and specificity for FGFR1 and β-klotho (140). The avimer showed FGF2-like activity, which was more potent than FGF21. It had a terminal half-life of 50 h after fusion with human serum albumin (C3201-HSA), and simulated the effects of FGF21 on obese monkeys, which lowered body weight. Due to the excellent pharmacokinetics and targeting specificity of monoclonal antibodies (mAbs), many research methods have been used to develop agonist mAbs for FGF21 receptor complexes. Two fully humanized FGF21-mimetic mAbs (mimAb1 and 39F7 mAb) bind to distinct conformational epitopes of β-klotho with high affinity and specifically activate cellular signaling via the FGFR1c–β-klotho complex. Injection of mimab1 in obese monkeys, resulted in FGF21 like metabolic effects, including body weight, plasma insulin, plasma triglyceride and glucose levels (141). *In vitro* characterization demonstrated that, 39F7 mAb is specific for β-Klotho/FGFR1c activation, but it does not compete with FGF21. Furthermore, the agonistic activity of 39F7 mAB required the full IgG molecule to be bivalent, suggesting that 39F7 functions by promoting β-Klotho/FGFR1c dimerization (142). Recently, Merck Sharp & Dohme is developed a monthly antibody MK-3655 (previous name NGM313) that activates the β-klotho-FGFR1c complex. A single-dose of MK-3665 can reduce liver fat content, improve dyslipidemia, and reduce HbA1c and transaminase in patients with obesity, insulin resistance and NAFLD (143) (Table 1).

**Table 1:** Pharmacological strategies to modulate the effects of FGF21 on related metabolic diseases.

**Brand name**	**Diseases/symptoms**	**Effects**
**FGF21 analogs**		
LY2405319	Obesity and T2DM (144, 145)	Dyslipidemia↓ Body weight↓ Plasma insulin↓
PF-05231023	Obesity with or without T2DM (134)	Serum TGs↓
Pegbelfermin (BMS-986036)	Obesity, T2DM, and NASH (135)	Dyslipidemia↓ Adiponectin ↑ The fibrosis biomarker N-terminal type III collagen propeptide (PRO-C3) ↓
Efruxifermin (AKR-001)	NASH and T2DM (146)	Liver fat↓ Body weight↓ Fibrosis↓
**FGF21-receptor agonists**		
C3201–HSA	Obesity and insulin resistance (147)	Body weight↓
MimAb1 and 39F7 mAb	Obesity (148)	Body weight ↓ Blood glucose and triglycerides ↓
MK-3655 (NGM313)	Obesity, insulin resistance, T2DM and NAFLD (143)	Liver fat↓ Dyslipidemia↓ HbA1c and transaminase↓

FGF21 analogs and FGF21RA tested in clinical trials are generally well-tolerated. However, PF-05231023 increased heart rate and blood pressure and caused moderate changes in bone resorption and resorption markers, which is consistent with the effect of FGF21-induced bone loss (139). Therefore, PF-05231023 improved the safety of FGF21 induced bone loss, which was observed in mice. Another side effect is the production of anti-fgf21 antibody caused by the immunogenicity of engineered FGF21, which was detected in more than 50% of patients treated with pegbelfermin and those treated with ly2405319(135). Of note, the duration of all the above clinical trials for FGF21 treatment is quite short (several weeks). Since FGF21 based treatment is targeted for chronic metabolic diseases, which often require lifelong medication, the safety issues related to long-term treatment need to be thoroughly examined in the future.

## Summary

Aging and metabolism are inextricably linked. With aging, metabolic function decreases, and metabolic homeostasis becomes unbalanced. Long-term metabolic imbalance will also accelerate cellular aging. Studies have found that steatotic hepatocytes always display severe DNA damage and cell cycle arrest, indicating that they have entered an aging state (149). Inducing aging cells *in vivo* and *in vitro* will disrupt the metabolic balance and promote excessive lipid deposition.

As a metabolism regulator, FGF21 can regulate the homeostasis of lipid and glucose metabolism and improve cellular aging (150, 151). AMPK, the downstream protein of FGF21, may be the key to FGF21's effect. AMPK plays a pivotal role in lipid metabolism. By upregulating the expression of ATGL, AMPK promotes lipolysis. In contrast, by regulating the expression of ACC1 and SREBP1c, AMPK can inhibit lipid synthesis. After being activated by FGF21, AMPK can also regulate the autophagy-related gene mTORC1 and the inflammation gene NF-κB to achieve anti-aging effects. AMPK can control the autophagy-related pathways SIRT1/PGC-1α, SIRT1, and its downstream aging-related genes P53, HIF-1α to prevent cellular aging. At present, some FGF21 analogs (LY2405319, PF-05231023, pegbelfermin, efruxifermin, etc.) and FGF21 receptor agonists (C3201–HSA, mimAb1, and 39F7 mAb, NGM313, etc.) have been confirmed by animal or clinical experiments to have an excellent ability to reduce weight and improve glucose and lipid metabolism. However, no clinical studies have clarified the efficacy of FGF21 analogs and receptor agonists to improve cell aging. This requires further in-depth research.

Overall, the above preclinical and clinical studies indicate that FGF21 has a vital role in alleviating dyslipidemia, lipid metabolism and glucose metabolism through different molecular pathways and/or target organs. FGF may soon become a key target for the treatment of aging-related metabolic diseases. Although FGF21 has received much attention and is frequently used in treating aging-related diseases, it is almost entirely involved in improving metabolism. There are few or no studies involving FGF21 for the treatment of aging-related metabolic diseases by improving aging. I hope this review, which summarizes many previous studies, can help promote more research on FGF21 in aging-related metabolic disorders.

## Author Contributions

JY, YN, and BH participated in drafting the manuscript. JC, ML, and MY provided technical assistance. ZC and BH revised the manuscript. All of the authors read and approved the final manuscript.

## Conflict of Interest

The authors declare that the research was conducted in the absence of any commercial or financial relationships that could be construed as a potential conflict of interest.
